# Endocan is a reliable biomarker during continuous renal replacement therapy

**DOI:** 10.1186/s13054-019-2585-4

**Published:** 2019-09-03

**Authors:** Maxence Hureau, Alexandre Gaudet, Nathalie De Freitas Caires, Erika Parmentier, Julien Poissy, Thibault Duburcq, Philippe Lassalle, Daniel Mathieu

**Affiliations:** 1 University Lille, U1019 – UMR 8204 – CIIL – Center for Infection and Immunity of Lille, F-59000 Lille, France; 2 CNRS, UMR 8204, F-59000 Lille, France; 3 INSERM, U1019, F-59000 Lille, France; 4 CHU Lille, Pôle de Réanimation, Hôpital Roger Salengro, F-59000 Lille, France; 5 Lunginnov, 1 rue du Pr Calmette, F-59000 Lille, France; 6 Institut Pasteur de Lille, F-59000 Lille, France

Dear Editor,

We read with attention the letter of Honoré et al. questioning the performance in diagnosis and prognosis of plasma endocan concentration in septic patients admitted in intensive care units with sepsis-associated acute kidney injury (SA-AKI) who have renal replacement therapy (RRT) [1]. Honoré et al. state that classical techniques with continuous RRT (CRRT) including high dose of separately filtration or dialysis as mixed techniques with hemofiltration, hemodialysis, and adsorptive treatment with highly adsorptive membranes (HAM) should decrease plasma concentration of endocan due to its 20-kDa protein core molecular weight [1].

In a strict structural point of view, endocan is circulating as a proteoglycan with an apparent molecular weight of 50 kDa by Western blot, and an average molecular mass of 400 kDa determined by gel filtration (GF), a direct consequence of its unique and linear dermatan sulfate chain of 15–40 kDa [2]. Thus, it seems unlikely that conventional CRRT having a 35-kDa membrane cut-off can remove endocan. However, we agree with Honoré et al. that new HAM could adsorb endocan, but until now, there is no data supporting this idea. Moreover, HAM is limited to highly selected patients with no clear benefit on mortality and should not affect the performance of endocan in current practice [3].

Cleaved endocan, called p14, is the major circulating catabolite of endocan generated by the neutrophil-derived cathepsin G. It corresponds to the 14-kDa N-terminal part of the protein core and deletion of the 6-kDa C-terminus bearing the glycanic chain. This catabolite contains 18 cysteine residues, conferring a highly rigid and globular structure [4]. Furthermore, a recent clinical investigation shows that plasmatic endocan cleavage ratio in septic patients increases with the severity state of SA-AKI [5]. This suggests that unlike endocan, p14 could be eliminated by the kidney and by CRRT.

Basically, endocan cannot be removed by CRRT, but doubt remains on HAM effect with endocan. By contrast, p14 could be eliminated through glomerular filtration in patients with preserved renal function, thus suggesting that it should be measured in urine rather than in blood. In patient with renal failure, p14 could be removed by CRRT and we do not know yet the reliability of its dosage in blood neither in urine. Therefore, endocan performances in diagnosis and prognosis should not be affected by CRRT. Further explorations are needed to confirm these hypotheses.

## Data Availability

Not applicable.
